# The Perceived Broad Group Emotional Climate Scale: Development and Validation With Chinese Community Residents and University Students

**DOI:** 10.3389/fpsyg.2021.686734

**Published:** 2021-08-25

**Authors:** Xiao-juan Yang, Xin-qiang Wang, Jian-ping Liu, Song-hai Lai, Mingfan Liu, Baojuan Ye

**Affiliations:** ^1^School of Psychology, Center for Mental Health Education and Research, Institute of Psychological Technology Application, Jiangxi Normal University, Nanchang, China; ^2^School of Marxism, Kunming Medical University, Kunming, China

**Keywords:** perceived broad group emotional climate, scale development, validity, residents, university students, social well-being, social attitude

## Abstract

Perceived broad group emotional climate (PBGEC) is a perceived meso-environment emotion, which refers to individuals’ perceptions and experiences of the emotion climate when interacting with group members in daily life, and is not derived from individuals’ own emotions. The purpose of this study was to develop and validate a PBGEC scale (PBGECS) for Chinese community residents and university students. A total of 1,408 residents from Chongqing completed the survey of PBGECS, the present social attitude scale, the future social expectations scale, and the social wellbeing scale, which constituted Sample 1; A total of 607 college students from Nanchang completed the survey of PBGECS and the Positive and Negative Affect Scale, which constituted Sample 2. Exploratory factor analysis revealed a two-factor structure, including positive PBGEC (PBGEC-P) and negative PBGEC (PBGEC-N). Internal consistency was strong for each factor and the full-scale (*α* ≥ 0.83). Confirmatory factor analysis showed that the correlated two-factor model of PBGEC and the four-factor model (including PBGEC-P, PBGEC-N, individual positive affect, and individual negative affect) demonstrated the best fit to the data, which supported the structural validity of the PBGECS. The interpretive validity, cultural validity, and population validity of the scale were also proved by examining the relationship between PBGEC and socioeconomic status, social attitude, and social wellbeing, respectively. The results show that the PBGECS demonstrated satisfactory reliability and validity, which can be used to assesses the perceived emotion climate of an individual’s surrounding environment.

## Introduction

Groups are “the natural environment of the mind” (Caporael and Baron, 1997). In other words, individuals’ emotional states and most aspects of their personality are fundamentally linked to group members who provide important information on how individuals can understand themselves and their environment. The perceived broad group emotional climate (PBGEC) guides their social attitude and social wellbeing (Keyes, 1998; Wang, 2011; Liu and Wang, 2020). Over the last 30 years, research on group emotions has made remarkable progress, and people are increasingly aware of the existence of group emotions (George, 1990, 1996; Barsade and Gibson, 1998; Kelly and Barsade, 2001; Collins et al., 2013; Barsade and Knight, 2015; Knight and Eisenkraft, 2015). Indeed, group emotions are no longer at the periphery of group and team research; it has increasingly become the center of this field (Barsade and Knight, 2015). However, few studies have considered group emotions as the individual’s meso-environment, or the external environment that connects individuals and groups—that is, the positive and negative forces that individuals experience in their interactions with others (Paquette and Ryan, 2001). Our focus in the present study is on this meso-environment emotion (De Rivera, 1977; De Rivera and Páez, 2007), which we refer to as PBGEC. The purpose of the current research was to develop and validate a theory-based and psychometrically sound measure that captures Chinese community residents and university students’ experiences of PBGEC, and examine preliminary associations between PBGEC and social attitude, social wellbeing correlates.

### Distinguishing Perceived Broad Group Emotional Climate From Related Concepts

General group emotions refer to emotions about group membership or belonging to the group (Kuppens and Yzerbyt, 2014). They have a “broader focus” (Smith et al., 2007). For example, people could feel proud to belong to a group if others view their group as prestigious (Kuppens and Yzerbyt, 2014). Group emotional climate refers to the predominant general group emotions generated through the social interaction of a group’s members in a particular milieu (De Rivera and Páez, 2007). For example, when a group wins, the emotional climate of the group is filled with joy and excitement. Compared with general group emotion, group emotion climate has a more dynamic quality of emotional construction. It is based on and shared with individuals through social interactions (De Rivera and Páez, 2007).

In this research, PBGEC is conceptually similar to group emotional climate, which exists as independent from an individual’s personal feelings (De Rivera, 1992; De Rivera and Páez, 2007; Páez et al., 2013) and reflects what individuals perceive regarding how most people feel about their ingroup’s situation (Páez et al., 2013). However, PBGEC and group emotional climate are different in perceived emotional subjects. PBGEC is not related to their own feelings but to that of their group members, for example, a bystander who can feel the group emotional climate of joy and excitement when a team wins. In that sense, “group emotional climate” is the same as “individuals” perceptions of the emotional climate.” According to the definition of PBGEC in this study, an individual perceives the emotional climate from the point of view of group members. This emotion is at the group level and has a broader focus, rather than being based on a particular event.

Currently, a question remains: What is the relationship between PBGEC and individual emotion? Can they both exist within a person at the same time? PBGEC and individual emotion appear to differ in both their theoretical underpinnings and in their experience. For example, people may perceive the broad group’s emotion as positive, but their own emotion as negative (Mackie and Smith, 2017). This suggests that PBGEC and individual emotion are independent constructs. PBGEC and individual emotion also have different influence ranges. That is, PBGEC can be shared, while individual emotion is owned by the individual. Numerous studies have shown that sharing emotion with others can lead to emotional convergence—that is, the phenomenon of emotion becoming consistent among group members (Totterdell et al., 1998; Totterdell, 2000; Barsade, 2002; Ilies et al., 2007; Wang et al., 2017). This sharing can, however, amplify individuals’ own emotional experiences. For example, watching a game with others may cause individuals to experience stronger emotions (e.g., excitement when one’s team wins the game) than when watching the game alone. Again, this amplification effect suggests that PBGEC and individual emotion are distinct. Thus, the emotional climates in countries, broad groups, or organizations are the result of social construction processes that are situated at the meso-level of the social interactions among individuals (Pelletier, 2018).

**Figure 1:**
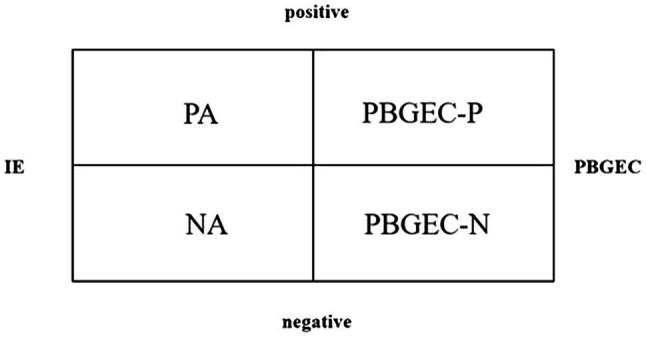
Four-factor diagram.

However, the precise mechanisms that underpin the construction of emotional climate remain rather unclear. This research demonstrates the existing link between personal emotions [measured specifically with the Positive and Negative Affect Schedule (PANAS); Watson et al., 1988] and PBGEC. We therefore predict that PBGEC and individual emotion will be distinct but related concepts. For Hypothesis 1, we predict that PBGEC and individual emotion will be related; i.e., the positive PBGEC (PBGEC-P) and negative PBGEC (PBGEC-N) will positively correlate with individual positive affect (PA) and negative affect (NA), respectively. In that, they will share the dimension of valence, but will focus on different subjects, and thus will form four groups (see Figure 1). In other words, we presume that PBGEC and individual emotion will conform to a four-factor model (PBGEC-P, PBGEC-N, PA, and NA).

### Defining Perceived Broad Group Emotional Climate

Climate research can focus on both individual and group levels (Florin et al., 1990). Climate includes emotion (De Rivera, 1992) and exists within social interactions (Schneider and Reichers, 1983). Emotional climate adds a dynamic quality, as it is based on and shared with individuals through social interactions, and is often quite stable (Bar-Tal, 2007; De Rivera and Páez, 2007; Páez et al., 2013; Bobowik et al., 2017). It refers to the emotional states perceived and experienced by the majority of group members (De Rivera and Páez, 2007). The concept may be useful in linking macro- and micro-levels of analysis (De Rivera and Páez, 2007).

Group emotional climates typically include the emotion and mood aspects of groups. We adopted a broad perspective, using the term “emotion” as it is used in everyday language to refer to affectively charged cognitions, feelings, moods, affect, and wellbeing (Bakhtiar et al., 2018). Our study examines the influence of broad group emotional climate on individuals and is not concerned with specific emotional changes in the individual. Accordingly, we do not distinguish between emotions and moods (Forgas, 1995, 2002; Beedie et al., 2005) and use the term “emotion” to refer to both emotions and moods.

In summary, PBGEC is a perceived meso-environment emotion, which refers to individuals’ perceptions and experiences of the emotion climate when interacting with group members in daily life, and is not derived from individuals’ own emotions. Specifically, PBGEC is derived from the people around the individual and not from the individual’s own emotions. It reflects individual perception of how most people feel about their ingroup’s situation (Páez et al., 2013), i.e., meso-environment. Although this kind of PBGEC is created by groups, it is felt by the individual. For example, what emotional experiences do you think people around you (such as those in your community or your classmates) have experienced? Thus, PBGEC can be shared and influences how people interact with the group, either directly or indirectly. In this way, PBGEC helps individuals adapt their positions and viewpoints in accordance with the group or meso-environment, and individuals further adapt to their social lives in general (Lai, 2013).

### Dimensionality of Perceived Broad Group Emotional Climate

An important dimension of subjective experience is valence; that is, something being pleasant or unpleasant (commonly referred to as positive or negative; Barrett and Russell, 1999). In their classification of emotion, Watson and Tellegen (1985) refined the valence concept using the terms positive emotion (PA) and negative emotion (NA). PA often reflects one’s level of happiness, passion, activeness, alertness, and other pleasant emotional states. In contrast, NA generally entails various unpleasant emotional states, including anger, contempt, disgust, guilt, fear, and nervousness (Watson et al., 1988; Gaudreau et al., 2006). Watson et al. (1999) observed the PA–NA dichotomy across a variety of cultural contexts which demonstrated good cross-cultural applicability.

In the present study of the PBGEC, we also use the dimensions of valence (positive–negative) to define the emotional climate. We define PBGEC-P as individuals’ perceptions and experiences of positive emotional climate, such as those of pleasure and enthusiasm, when interacting with group members in daily life. In contrast, PBGEC-N is defined as individuals’ perceptions and experiences of negative emotional climate, such as those of anxiety and worry, when interacting with group members in daily life.

To provide a solid basis for investigating the distinction between PA and NA (e.g., through PANAS, Watson et al., 1988), most researchers have proposed two-factor models for their measurements (i.e., uncorrelated or correlated two-factor models). For instance, Watson et al. (1988) observed, based on an exploratory factor analysis (EFA), that the PANAS comprises two orthogonal factors. Other studies have reported that correlated two-factor models exhibit significantly improved fit compared to single-factor and uncorrelated two-factor models (Seib-Pfeifer et al., 2017). Based on the above-discussed classifications of emotion and emotional climate, we suggest that the valence (positive–negative) dimension can be used to jointly classify PBGEC. Accordingly, we speculate that the PBGEC scale (PBGECS) will have a similar structural model to the PANAS. Given this, our Hypothesis 2 is that the PBGECS will have both positive and negative dimensions, and will thus conform to a two-factor model.

### The Effect of Socioeconomic Status on Perceived Broad Group Emotional Climate

Emotional climate depends on economic and educational factors and may change within the course of a single generation (De Rivera, 1992) due to socioeconomic status (SES), usually measured *via* education level and household income (Mahdavian and Safizadeh, 2015). In other words, people of different SES are likely to face different meso-environments and social mentalities (Liu and Wang, 2020). Thus, individuals with a higher SES, indicating greater wealth, tend to feel more positive emotional climates (De Rivera et al., 2007). For example, the underclass people report significantly less trust and security and more anger and fear than working-class and middle-class people (De Rivera et al., 2007). More specifically, with the development of online shopping, some educated online shoppers (from the middle-class) may perceive a PBGEC-P around them as most of their middle-class neighbors or colleagues enjoy the convenience of online shopping; other uneducated off-line shoppers (from the working-class) may feel a PBGEC-N around them as most of their working-class neighbors or colleagues feel abandoned in the era of online payments. It is valuable to understand the relationship between participants and their cultures (Altheide and Johnson, 1994). Previous studies have found that there was a significant negative correlation between SES and negative emotions (e.g., depression and anxiety; Salami and Walker, 2014), and a significant positive correlation between early-life SES and state positive affect (e.g., relaxed, cheerful, and pleased; Murdock et al., 2017). Therefore, PBGEC may be influenced by SES. The present study will examine associations with PBGEC to test the external validity of the scale. For Hypothesis 3a, we hypothesize that SES will have a significant positive influence on PBGEC, with individuals who have a high SES scoring higher in PBGEC-P than those who have a low SES. For Hypothesis 3b, we propose that individuals who have a low SES will score higher on PBGEC-N than those who have a high SES.

### The Function of Perceived Broad Group Emotional Climate

Individuals adjust their standpoints and viewpoints *via* direct or indirect interactions and experiences with group members, which in turn affect their emotions and attitudes toward events (Lai, 2013). In a similar way, PBGEC, by virtue of its existence in the meso-environment, leads to the formation of perceptions that affect individuals’ attitudes, motivations, and behaviors (De Rivera and Páez, 2007).

Existing research shows that group emotions can influence the attitude of individuals (Frijda and Mesquita, 1994; Niedenthal and Brauer, 2012). For example, negative group emotions (such as anger and disgust) can reduce individual group members’ contact with others outside of the group (Esses and Dovidio, 2002; Lu et al., 2016). In contrast, group positive emotion (e.g., gratitude) can reduce group members’ bias against outgroup members (e.g., prejudice; Miller et al., 2004; Lu et al., 2016). Therefore, PBGEC may also influence individuals’ attitudes.

Ma (2010) designed a social attitude questionnaire to assess present social attitudes and future social expectations. Present social attitudes refer to an individual’s feelings regarding group members in the social environment (Ma, 2010) and the state in which they live, both of which can positively or negatively influence a person’s social judgments (Cook and Bird, 2011). According to the value expectation theory of Atkinson and Cartwright (1964) and the future orientation theory of Nurmi (1991), the future social expectations refer to an individual’s judgment of his or her social future development and living environment that is based on the individual’s current situation. Individuals’ perceptions of the social status quo are related to their expectations of the future of a society (Chen et al., 2018). To investigate the relationship between PBGEC and individuals’ social attitudes and expectations, we propose a Hypothesis 4; that is, PBGEC will be related to individuals’ present social attitudes and future social expectations. Specifically, the PBGEC-P will positively correlate with a positive present social attitude and positive future social expectations; the PBGEC-N will be associated with a negative present social attitude and negative future social expectations.

Group positive and negative emotions influence wellbeing (Smith et al., 2007; Gamero et al., 2008). In the present study, we also examined how PBGEC influences wellbeing. Social wellbeing (Keyes, 1998) focuses on the perceived quality of individuals’ social relations and fulfillment of social tasks. This suggests that social wellbeing should be correlated with PBGEC. Therefore, we investigate this in Hypothesis 5.

### Perceived Broad Group Emotional Climate Measurement

Perceived broad group emotional climate is considered to be a latent construct that cannot be directly observed and is therefore difficult to measure objectively (Marraccini et al., 2020). Studies have discussed how to measure the emotional climate of organizations (Ruiz, 2007). They either measure the role that emotions play in the organization or measure one aspect of emotional climate in the organization (Ruiz, 2007). In the present study, we regard accounting for individuals’ subjective experiences as the most important aspect to consider when assessing PBGEC.

### Overview of the Present Study

This study sought to develop a PBGECS and assess its validity and reliability. In addition to analyzing the structural validity of the scale, this study also examined the cultural, population, and interpretive validity of the scale based on the indicators of cultural understanding described by Washington and McLoyd (1982). Specifically, interpretive validity, which includes the voices of the participants, is assessed by SES (Mueller and Parcel, 1981). Cultural validity is represented by the use of the Social Attitude Scale (Ma, 2010). This scale assesses attitudes toward the present and the future of China. Population validity is assessed by the use of the Social Well-Being Scale (Zhao, 2010). In general, the hypotheses are summarized as follows: (1) PBGEC relates to, but differs from, individual emotion; PBGEC, individual emotion, and valence form a four-factor model (PBGEC-P, PBGEC-N, PA, and NA); (2) PBGEC has two dimensions: PBGEC-P and PBGEC-N; (3) SES significantly influences PBGEC: individuals with a high SES will score higher on the PBGEC-P (Hypothesis 3a) and individuals with a low SES will score higher on PBGEC-N (Hypothesis 3b); (4) PBGEC correlates with individual perceptions of present social attitudes and future social expectations; and (5) a correlative relationship exists between PBGEC and social wellbeing.

## Materials and Methods

Currently, there is a lack of a PBGEC measurement scale that enables us to assess how individuals evaluate the emotional climate of their groups. Thus, we compiled a questionnaire of adjectives most frequently used to describe broad group emotions perceived by individuals in daily life. By combining theoretical analysis with empirical investigation, we developed the PBGECS. Reliability and validity of PBGECS were subsequently tested in order to provide an effective, practical, and simple evaluation tool for measurement of PBGEC.

In general, group emotions are studied using one of two methods (Smith et al., 2007): The first method is to study people’s emotional reactions to specific objects or events, and the second is to let people express their own emotional perceptions. We emphasized that PBGEC is derived from the people around them and not from the individual’s own emotion. Thus, in the present study, we used the latter method; that is, we asked participants to describe how they perceived the broad group emotions of the people in their immediate living environment. For example, “*To what extent do you feel these (given) descriptive (positive/negative) emotional words are experienced by people around you?*” Participants then rated the words on a 5-point Likert scale with answers ranging from 0 (*not at all*) to 4 (*very strongly*).

We used different instructions to test the participants’ individual emotions. For example, “*This scale consists of a number of words that describe different feelings and emotion. Read each item and then mark the appropriate answer in the space next to that word. Indicate to what extent you have felt this way during the past week. Use the following scale to record your answers*.” Answers to the PANAS items range from “*not at all*” to “*very strongly*.”

### Item Generation

Based on the previous research, the present study surveyed PBGEC among community residents and university students in accordance with prior theoretical assumptions. At the same time, in considering the characteristics of Chinese people’s emotional experience and expression, we did not use an arousal or activity dimension when structuring the PBGEC (Liu et al., 2014); rather, we directly used positive and negative dimensions.

In step 1, with reference to the PANAS (particularly its expanded list of positive and negative adjectives), phrases that did not duplicate PANAS items (but still reflected PA and NA concepts) were selected from the Circumplex Model of Affect (Russell, 1980; Watson and Tellegen, 1985; Barrett and Russell, 1999). This model found that the fundamental affect was the similarity between English- and Chinese-speaking respondents (Yik and Russell, 2003). Next, we selected certain words for the item pool. Examples include strong, excited, enthusiastic, proud, vibrant, afraid, uneasy, distressed, jittery, and anxious.

In step 2, we integrated this information with the 12 emotion adjectives that were investigated by Smith and colleagues (Smith et al., 2007; Moons et al., 2009). All words were divided into positive and negative PBGEC indicators. Examples include trust, afraid, indifferent, indignant, enthusiastic, afraid, enjoyable, safe, cheerful, happy, misery, and worried.

In step 3, frequently used phrases based on participant reports were extracted from a social emotional climate glossary published by Wang (2011, 2013a,b,c, 2018). In 2006, Wang and his colleagues completed the largest survey of social mentality in China and conducted a comprehensive study on the current social mentality of Chinese people (Wang et al., 2007). They identified the core set of Chinese group emotional climate, including anxiety, indifference, indignation, misery, cheer, blunder, and greed. The anxiety set includes words, such as uneasy, worried, afraid, and fearful. Indifference includes accidie, disregard, unconcerned, and unmoved emotions; indignant is caused by dissatisfaction, hostility, resentment, animosity, and other degrees of anger; misery is caused by life pressure, unemployment, family unhappiness, disasters, accidents, and other negative social emotions. Cheerful reflects the overall happiness of social members, including hopeful, enthusiastic, happy, excited, vibrant, inspiring, grateful, and satisfied. Blundering is a social irrationality caused by many complex reasons. Greed is the emotional expression of excessive social needs (Wang, 2013a).

These selected words were used as references during scale development. In addition, Chinese society advocates the creation of climates with such attributes as fair, coherent, free, delighted, and so on. After repeated rounds of discussion and expert reviews, a pilot PBGECS, which included 39 words describing positive and negative emotional experiences, was developed. Scale validity and reliability were tested to determine whether this scale would be an acceptable PBGEC measure.

### Participants

#### Sample 1

We adopted a stratified random sampling strategy from 13 counties and 3 high schools from a municipality of Chongqing in Southwest China. From there, a unified testing method was implemented. The questionnaire was completed anonymously and was returned to the research team immediately following completion. In this process, participants first indicated that they understood our research purpose, process, matters needing attention, and confidentiality principle, and then filled in the informed consent. We then invited subjects to complete questionnaires. Finally, each participant received a reward of a small gift. A total of 1,519 questionnaires were distributed, and 1,408 valid questionnaires were returned (effective recovery rate = 92.7%). Participants’ sociodemographic characteristics are summarized in Table 1.

**Table 1:** The sociodemographic characteristics of participants.

Topic	Sample	Topic	Sample
**Gender**		Not provided	1.0%
Male	46.6%	**Age**	
Female	45.3%	17–25	27.4%
Not provided	8.1%	26–35	29.9%
**Marital status**		36–45	25.1%
Unmarried	33.1%	46–55	13.6%
Married	60.3%	More than 56	2.3%
Divorced	4.8%	Not provided	1.8%
Widowed	0.6%	**Educational level**	
Not provided	1.1%	JH	9.9%
**Occupational backgrounds**		H/T	18.3%
Administrators	3.6%	C	22.8%
Business managers	4.9%	U	41.3%
Private entrepreneurs	3.7%	M & D	7.5%
Professional technicians	8.5%	Not provided	0.3%
Staff from party government offices/companies/institutions	23.2%	**Household income**	
Individual industrial and commercial workers	8.1%	Less than 10	8.2%
Business services personnel	6.0%	10–20	10.7%
Manual workers	2.6%	20–40	22.9%
Farmers	6.4%	40–60	23.1%
Full-time students	19.5%	60–100	17.5%
Unemployed individuals	2.7%	100–150	7.9%
Emeritus and retired	0.4%	150–200	5.0%
Freelancers	5.0%	More than 200	4.2%
Others	4.5%	Not provided	0.5%

*N_sample 1_ = 1,408. Educational level: JH, Junior high school level or below; H/T, high school/technical secondary school level; C, college level; U, university level; M & D, Master’s degree and doctor’s degree level. Household income:1,000 RMB is the unit of measurement*.

#### Sample 2

To determine whether PBGEC and individual emotion are different emotional constructs, the present study employed the convenient sampling strategy by recruiting students from five general elective classes at a university in Nanchang, Jiangxi Province, Central China. Participants were from different departments and in different years in university, and were approximated by random sampling. The investigation process was the same as for Sample 1. Each participant received a small gift in return for their participation. A total of 700 questionnaires were distributed, and 607 valid questionnaires were returned (effective recovery rate = 86.79%). Of the 607 participants, 21.9% were male, and 78.1% were female, which is consistent with the gender distribution of college students in this school. There was no significant difference in gender. The results of the *t*-test were −0.32 (*p_PA_* = 0.75), 0.06 (*p_NA_* = 0.95), −0.02 (*p_PBGEC-p_* = 0.99), and −1.80 (*p_PBGEC-N_* = 0.07), respectively. Age proportions were as follows: <20 years (75.3%), 20–25 (24.2%), and 26–35 (0.5%). There was no significant difference in age too. The results of ANOVA were 0.10 (*p_PA_* = 0.91), 1.58 (*p_NA_* = 0.21), 0.04 (*p_PBGEC-p_* = 0.96), and 1.57 (*p_PBGEC-N_* = 0.21), respectively.

#### Sample 3

After the PBGECS was developed, 102 participants (78 females; 7 residents and 95 college students, aged between 18 and 58 years, and recruited from three universities in Jiangxi Province and Yunnan Province in China) were followed up online for 1 month.

### Measures

In the present study, we draw lessons from the Cronbach and Meehl’s (1955) classic structural validation proposal to explore the internal structure of the scale, establish the test–retest reliability, and determine the criterion validity of the scale. For Sample 1, we developed a general information questionnaire to collect data pertaining to demographic characteristics, including gender, age, marital status, education level, household income, and occupation. For Sample 2, we only collected data pertaining to demographic characteristics, including gender and age.

### Self-Generated PBGECS-39 Items Pilot Questionnaire

The perceived broad group emotional climate scale-39 (PBGECS-39) is comprised 39 emotional adjectives, of which 19 items are positive emotional adjectives (e.g., cheerful, hopeful, and enthusiasm) and 20 items are negative emotional adjectives (e.g., anxious, uneasy, and worried). Participants were instructed to answer the following question for each adjective: “*To what extent do you think the people around you (such as your community residents or your classmates) experience the emotions described by the following words?*” The participants responded based on a 5-point Likert scale with answers ranging from 0 (*not at all*) to 4 (*very strongly*).

### Positive and Negative Affect Schedule

We adopted the Chinese version of the PANAS (Huang et al., 2003) to measure individual emotion. Huang et al. (2003) conducted a study on the applicability of PANAS in a Chinese population and found that Cronbach’s *α* reliability coefficients for a Chinese population for positive and negative emotion was 0.88 and 0.85, respectively. This shows that the Chinese version, like the English version, has a high degree of homogeneity. In our study, the PANAS was also comprised two dimensions: PA (10 items) and NA (10 items). The participants responded based on a 5-point Likert scale with answers ranging from 0 (*not at all*) to 4 (*very strongly*). In the present study, Cronbach’s *α* reliability coefficients were 0.94 and 0.93 for the PA and NA subscales, respectively.

### Present Social Attitude Scale

For the present study, we employed the social attitude scale developed by Ma (2010). Using this scale, it is possible to only administer specific subscales. Thus, we employed the present social attitude subscale and the future social expectations subscale. The present social attitude scale consists of 10 items and was used to measure participants’ present social attitudes. Responses were provided on a Likert-type scale ranging from 1 (*strongly disagree*) to 6 (*strongly agree*). The scale consists of two subscales: a positive present social attitude subscale, which is comprised 5 items (e.g., “*China’s current political and economic condition is good*”), and a negative present social attitude subscale, which is also comprised 5 items (e.g., “*overall, the general social conduct is not as good as before*”). In the present study, the Cronbach’s *α* reliability coefficient for the positive subscale was 0.80 (*p* < 0.001) and 0.72 (*p* < 0.001) for the negative subscale.

### Future Social Expectations Scale

A 4-item scale developed by Ma (2010) was implemented to investigate participants’ future social expectations (Chen et al., 2018). A Likert-type response scale ranging from 1 (*strongly disagree*) to 6 (*strongly agree*), including two subscales, was used. The first is a positive future social expectations subscale that comprises two items (e.g., “*I have full confidence in the future of China*”). The two positive expectation items were significantly correlated (*r* = 0.63, *p* < 0.001) in the present study. The second subscale, negative future social expectations, also comprises two items (e.g., “*Most people are not optimistic about the future of China*”). The two negative expectation items were also significantly correlated (*r* = 0.63, *p* < 0.001) in the present study.

### Social Well-Being Scale

Zhao (2010) developed the social wellbeing scale based on Keyes’ (1998) five-dimensional structure model. The scale comprises 22 items and five dimensions: social integration (four items, e.g., “*I feel that I belong to the community and society*”), social acceptance (four items, e.g., “*I feel comfortable with others*”), social contribution (five items, e.g., “*I believe that I am a vital member of society, with something of value to give to the world*”), social actualization (four items, e.g., “*I can envision that we like our community, and there are potential beneficiaries for social growth*”), and social coherence (five items, e.g., “*I feel that my life is meaningful and coherent*”). A Likert-type response scale ranging from 1 (*strongly disagree*) to 6 (*strongly agree*) was used. The Cronbach’s *α* reliability coefficients for each dimension were 0.77, 0.81, 0.76, 0.82, and 0.77, respectively. Internal consistency for the full-scale was 0.82 (*p* < 0.05).

### Procedure

Before data collection began, the research team was uniformly trained. The study followed principles of confidentiality and voluntary participation. All participants provided informed consent and completed the questionnaire anonymously and independently. For community residents (Sample 1), the basic sampling unit was a household. For university students (Sample 2), cross-disciplinary and cross-grade general elective classes were the primary sampling units. We used a combination of centralized and distributed surveys, and the questionnaires were collected on the spot.

Sample 1 was examined using the PBGECS-39, the present social attitude scale, the future social expectations scale, the social wellbeing scale, and the general information questionnaire. Sample 2 was examined using the PBGECS-20 (the revision of PBGECS-39, Appendix A), PANAS, and general information questionnaire (only gender and age). Sample 3 was examined using the PBGECS-39 (including the PBGECS-20). The interval between the two surveys was about 1 month, and the test–retest reliability of these two scales was obtained.

### Statistical Analyses

Statistical analyses were performed using SPSS 22.0 and Mplus7 (Muthén and Muthén, 1998–2012). SPSS 22.0 was used to analyze descriptive statistics and to perform correlation analyses. Mplus7 was used to perform a confirmatory factor analysis (CFA). According to the operating principle of factor analysis (Hu and Mo, 2002), Sample 1 (*N*_1_ = 1,408) was randomly divided into two subsamples (Sample 1A and Sample 1B), both with a sample size of 704. Data from Samples 1A and 1B were used to conduct an EFA and CFA, respectively. Sample 1 data also underwent reliability testing and criterion validity analysis. The data from Sample 2 was used to assess relations between the PBGECS and PANAS, and was then subjected to a CFA to evaluate the models of the relations between PBGEC and individual emotion.

## Results

### Item Analysis

The present study used a project differentiation analysis to check whether scale items were adequate. Total correlations and critical ratios (critical point at 27%) among items were used as analytical indices for item discrimination. Results from Sample 1 (Table 2) revealed that the correlation coefficients between the 39 PBGECS items ranged from 0.69 to 0.85, which indicates satisfactory item discrimination.

**Table 2:** Corrected item-total correlation and critical ratio for the PBGECS-39.

Item	Corrected item-total correlation (*r*)	Critical ratio (*t*)	Item	Corrected item-total correlation (*r*)	Critical ratio (*t*)	Item	Corrected item-total correlation (*r*)	Critical ratio (*t*)
愉悦 (Cheerful)	0.58	22.01	欣喜 (Joy)	0.56	20.63	漠然 (Accidie)	0.65	27.89
希望 (Hopeful)	0.55	22.12	和谐 (Harmonious)	0.60	23.05	不满 (Dissatisfied)	0.68	31.62
热情 (Enthusiastic)	0.60	23.93	满意 (Satisfied)	0.65	25.93	无奈 (Helpless)	0.64	26.71
开心 (Happy)	0.59	22.13	安全 (Safe)	0.61	24.73	失望 (Disappointed)	0.66	27.87
享受 (Enjoyable)	0.53	18.57	信任 (Trusting)	0.64	25.05	怨恨 (Resentment)	0.61	23.72
坚强 (Strong)	0.49	19.05	剬平 (Fair)	0.67	26.79	愤恨 (Indignant)	0.57	21.56
兴奋 (Excited)	0.55	20.34	焦虑 (Anxious)	0.61	22.70	敌视 (Hostile)	0.54	20.23
活跃 (Vibrant)	0.55	19.54	不安 (Uneasy)	0.63	23.94	仇恨 (Animosity)	0.51	19.22
鼓舞 (Inspiring)	0.56	20.37	担忧 (Worried)	0.63	24.57	痛苦 (Misery)	0.59	23.87
感激 (Grateful)	0.59	21.77	害怕 (Afraid)	0.61	24.36	压力 (Stressful)	0.55	22.24
自豪 (Proud)	0.57	21.22	恐惧 (Fearful)	0.57	22.03	浮躁 (Blundering)	0.59	24.42
自由 (Free)	0.54	19.34	郁闷 (Distressed)	0.62	24.81	贪欲 (Greedy)	0.62	26.46
正义 (Justicial)	0.60	23.54	冷漠 (Indifferent)	0.67	29.23	战战兢兢 (Jittery)	0.52	19.09

*N_sample 1_ = 1,408. PBGECS-39, perceived broad group emotional climate scale-39 items*.

### Factor Analysis

#### PBGECS-39 Factor Analysis

In Hypothesis 5, we predicted that the PBGEC would have two dimensions, positive and negative, and its structure would be a two-factor model. Therefore, our study used factor analysis to explore and verify the dimensions and structure of our scale, and to test whether the actual model was consistent with the theoretical model.

We randomly divided Sample 1 into two sections according to the operating principle of factor analysis (Hu and Mo, 2002). One section was used for the EFA, and another was used for the CFA. To verify if the data set was suitable for an EFA, the Kaiser-Meyer-Olkin (KMO) test and Bartlett’s test of sphericity were first performed on Sample 1A. Results revealed that *χ*^2^ = 22348.07, *p* < 0.001, KMO = 0.96, which indicated suitability for a factor analysis. Results from the Bartlett’s test of sphericity (*p* = 0.000) indicated the existence of a common factor within the correlation matrix, also suggesting suitability for an EFA. By using principal components analysis for the EFA and performing a varimax rotation, the analysis revealed five factors with eigenvalues greater than 1. However, the number of one item loaded on more than one factor at the same time is too much. From the previous literature, we can see that PBGEC has two factors (positive and negative). Therefore, we use the method of fixed factor number, and the factor extraction number is set to 2. Thus, 58.27% of the total variance was explained (see Table 3). According to the scree plot, the point of inflexion was at the second factor. Hence, it was reasonable to delineate two factors. The first factor referenced positive emotion; hence, this factor was termed “positive of perceived broad group emotional climate” (PBGEC-P). This factor’s eigenvalue (after rotation) was 11.42, with a contribution rate of 29.28%. The second factor referenced negative emotion; hence, this factor was termed “negative of perceived broad group emotional climate” (PBGEC-N). This factor’s eigenvalue (after rotation) was 11.31, with a contribution rate of 28.99%. The correlation coefficient between PBGEC-P and PBGEC-N was −0.33 (*p* < 0.001). The factor loading matrix is shown in Table 3.

**Table 3:** Factor loading matrix for exploratory factor analysis of the PBGECS-39.

Item	Factor 1	Factor 2	Communalities	Item	Factor 1	Factor 2	Communalities
**PBGEC-P Subscales**				**PBGEC-N Subscales**			
愉悦 (Cheerful)	−0.14	**0.73**	0.55	焦虑 (Anxious)	**0.72**	−0.16	0.54
希望 (Hopeful)	−0.10	**0.72**	0.53	不安 (Uneasy)	**0.74**	−0.17	0.57
热情 (Enthusiastic)	−0.13	**0.78**	0.63	担忧 (Worried)	**0.70**	−0.20	0.53
开心 (Happy)	−0.09	**0.80**	0.65	害怕 (Afraid)	**0.77**	−0.10	0.60
享受 (Enjoyable)	−0.09	**0.74**	0.55	恐惧 (Fearful)	**0.76**	−0.06	0.57
坚强 (Strong)	−0.04	**0.67**	0.45	郁闷 (Distressed)	**0.75**	−0.14	0.59
兴奋 (Excited)	−0.05	**0.80**	0.64	冷漠 (Indifferent)	**0.77**	−0.16	0.61
活跃 (Vibrant)	−0.05	**0.78**	0.62	漠然 (Accidie)	**0.76**	−0.17	0.60
鼓舞 (Inspiring)	−0.09	**0.78**	0.62	不满 (Dissatisfied)	**0.77**	−0.22	0.64
感激 (Grateful)	−0.15	**0.77**	0.61	无奈 (Helpless)	**0.70**	−0.26	0.55
自豪 (Proud)	−0.13	**0.77**	0.61	失望 (Disappointed)	**0.79**	−0.20	0.66
自由 (Free)	−0.15	**0.71**	0.52	怨恨 (Resentment)	**0.79**	−0.11	0.63
正义 (Justical)	−0.18	**0.75**	0.59	愤恨 (Indignant)	**0.78**	−0.04	0.60
欣喜 (Delighted)	−0.03	**0.82**	0.67	敌视 (Hostile)	**0.76**	−0.01	0.59
和谐 (Coherence)	−0.14	**0.72**	0.54	仇恨 (Animosity)	**0.74**	0.01	0.54
满意 (Satisfied)	−0.18	**0.81**	0.69	痛苦 (Misery)	**0.78**	−0.05	0.60
安全 (Safe)	−0.20	**0.72**	0.56	压力 (Stressful)	**0.59**	−0.18	0.38
信任 (Trust)	−0.22	**0.76**	0.62	浮躁 (Blundering)	**0.71**	−0.17	0.53
剬平 (Fair)	−0.28	**0.75**	0.64	贪欲 (Greedy)	**0.75**	−0.17	0.60
				战战兢兢 (Jittery)	**0.73**	−0.02	0.53

*N_sample 1A_ = 704. PBGECS-39, perceived broad group emotional climate scale-39 items; PBGEC-P, positive perceived broad group emotional climate; PBGEC-N, negative perceived broad group emotional climate. Factor loadings >0.40 are in boldface. Extraction method: Principal Component Analysis. Rotation Method: Varimax. Rotation converged in 100 iterations*.

Sample 1B data were examined *via* a CFA to assess scale construct validity. The EFA revealed that the PBGECS has a two-dimensional structure, supporting our hypothesis that the PBGEC can be classified into two dimensions: PBGEC-P and PBGEC-N. However, the degree of fit between theoretical constructs and the actual collected data required confirmation. CFA results (see Table 4) indicated that the uncorrelated two-factor model and the correlated two-factor model had a TLI and CFI over 0.80 (but lower 0.90) and a RMSEA under 0.08. Hence, these two-factor models indicated better fit than the one-factor model. Even though fit indices for both models reached a satisfactory level, these values were not ideal.

**Table 4:** Model fit indices for the PBGECS-39 and the PBGECS-20.

CFA model	*χ^2^*	*df*	SRMR	TLI	CFI	RMSEA	90% CI
**Sample 1B (*N*_sample 1b_ = 704) Original Scale (Items-39)**							
Model 1: one-factor	13300.40^***^	702	0.23	0.39	0.42	0.16	0.157–0.162
Model 2: uncorrelated two-factor	3550.31^***^	702	0.11	0.80	0.81	0.08	0.073–0.078
Model 3: correlated two-factor	3521.09^***^	701	0.07	0.80	0.81	0.08	0.073–0.078
**Shortened Scale (Items-20)**							
Model 4: one-factor	4484.85^***^	170	0.21	0.49	0.55	0.19	0.185–0.195
Model 5: uncorrelated two-factor	988.16^***^	170	0.13	0.90	0.91	0.08	0.078–0.088
Model 6: correlated two-factor	930.28^***^	169	0.05	0.91	0.92	0.08	0.075–0.085
**Sample 2 (*N*_sample 2_ = 607) Shortened Scale (Items-20)**							
Model 7: one-factor	3525.07^***^	170	0.19	0.51	0.56	0.18	0.175–0.186
Model 8: uncorrelated two-factor	1358.02^***^	170	0.10	0.83	0.85	0.11	0.102–0.113
Model 9: correlated two-factor	1334.68^***^	169	0.06	0.83	0.84	0.11	0.101–0.112

*PBGECS-39, perceived broad group emotional climate scale-39 items; PBGECS-20, perceived broad group emotional climate scale-20 items*.

*** *p < 0.001*.

#### PBGECS-20 CFA

We observed that the fit of several items did not reach the ideal level. Thus, the items were eliminated through a stepwise method, using the following standard: If removal of an item significantly increased overall scale reliability, the item was permanently eliminated. This procedure led to the retention of 20 emotion words. The 10 PBGEC-P words were cheerful, enthusiastic, happy, enjoyable, excited, vibrant, inspiring, proud, joy, and satisfied, while the 10 PBGEC-N words were anxious, worried, afraid, fearful, dissatisfied, distressed, misery, indifferent, resentment, and greedy.

**Figure 2:**
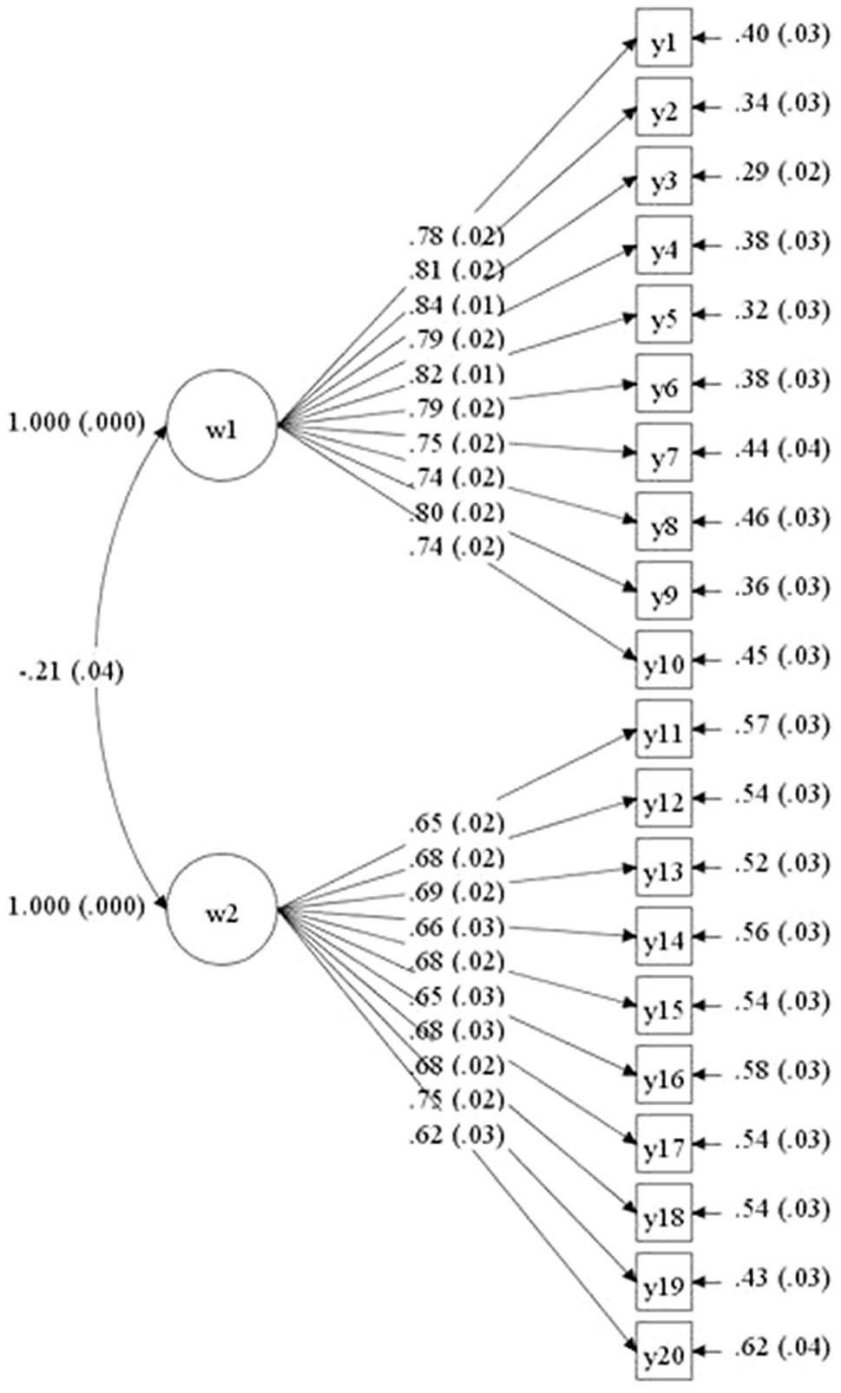
Corrected two-factor model for the perceived broad group emotional climate (PBGEC).

To verify the goodness of fit of the PBGECS-20, we tested the one-factor, uncorrelated two-factor, and correlated two-factor models for Sample 1B and Sample 2 (Figure 2). The CFA results suggested that the fit indices for the one-factor model were not ideal; in contrast, the correlated two-factor model had adequate fit (see Table 4). This indicated that the PBGECS-20 was more effective than the PBGECS-39 for measuring PBGEC.

In order to verify Hypothesis 1, we first investigated the relationship between PBGEC and individual emotion. Subsequently, we used CFA to explore whether PBGEC and individual emotion contain two factors (positive and negative) or four factors (PBGEC-P, PBGEC-N, PA, and NA).

We matched PBGEC and individual emotion data from Sample 2 to analyze the correlations between PBGEC and individual emotion. The results revealed that PA was positively associated with PBGEC-P (*r* = 0.58, *p* < 0.001), NA was positively associated with PBGEC-N (*r* = 0.54, *p* < 0.001), and PBGEC-P was negatively associated with PBGEC-N (*r* = −0.20, *p* < 0.001) and NA (*r* = −0.14, *p* < 0.05). The correlation coefficients *r* between PBGEC-N and PA and between PA and NA are not significant, which are 0.03 (*p* = 0.403) and −0.01 (*p* = 0.911), respectively.

### CFA of the PBGEC and Individual Emotional Relationship Model

**Figure 3:**
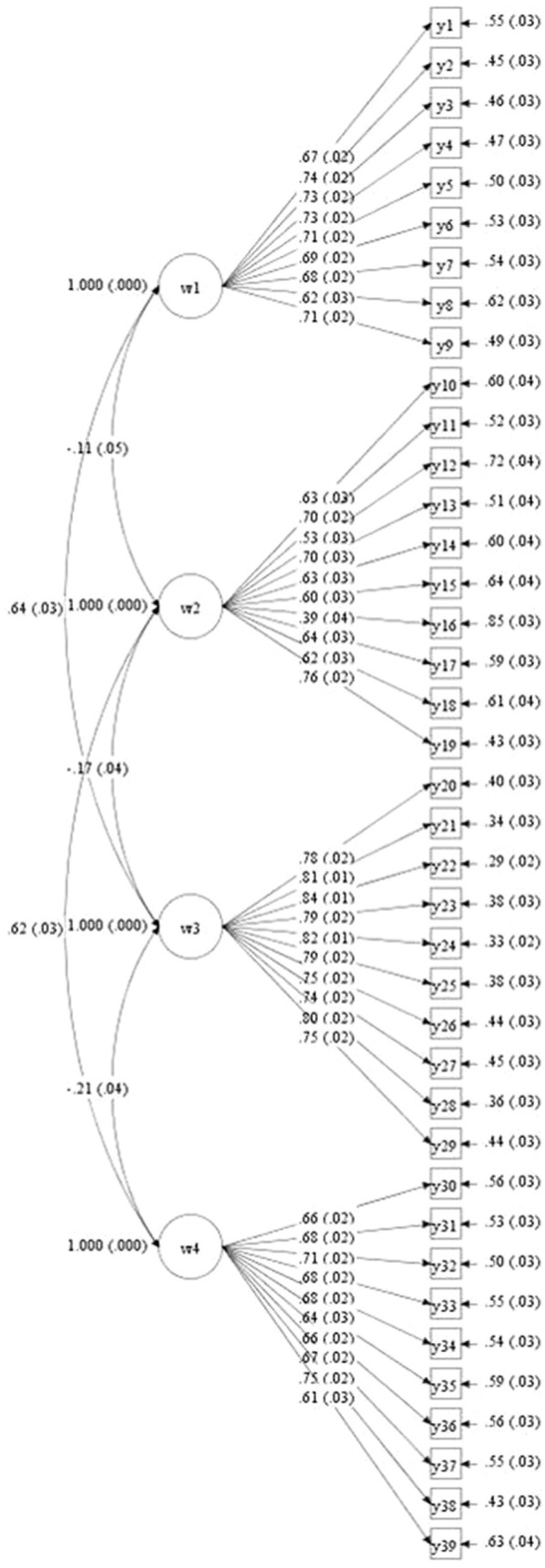
Four-factor model for the PBGEC and individual affect.

Using CFA, we assessed the fit of the one-factor model, two-factor model, and four-factor model. The results are shown in Table 5. The fit of the four-factor model was significantly better than the one-factor model and two-factor model (see Figure 3), suggesting that the PBGEC is different from individual emotion. The one-factor model was a pure measurement model, including all the measurement factors. The two-factor model included PBGEC and individual emotion, or positive and negative emotion. The four-factor model included PBGEC-P, PBGEC-N, PA, and NA.

**Table 5:** Model fit indices for PBGEC and the individual affect model.

CFA model	*χ^2^*	*df*	SRMR	TLI	CFI	RMSEA	90% CI
One-factor model	8045.65^***^	702	0.17	0.41	0.44	0.13	0.129–0.134
Two-factor model (PBGEC and individual emotion)	6911.41^***^	701	0.17	0.50	0.53	0.12	0.118–0.123
Two-factor model (positive and negative emotion)	4641.35^***^	701	0.08	0.68	0.70	0.10	0.094–0.099
Four-factor model (PBGEC-P, PBGEC-N, PA, and NA)	2709.70^***^	696	0.06	0.84	0.85	0.07	0.066–0.072

*N_sample 2_ = 607*.

*PBGEC, perceived broad group emotional climate; PBGEC-P, positive perceived broad group emotional climate; PBGEC-N, negative perceived broad group emotional climate; PA, positive affect; NA, negative affect*.

*** *p < 0.001*.

### Validity and Reliability for the PBGECS-20

#### Reliability

Internal consistency on the PBGECS-20 revealed that for Sample 1, Cronbach’s *α* for the full-scale was 0.83 (*p* < 0.001). The PBGEC-P scale had a Cronbach’s *α* = 0.94 (*p* < 0.001), the composite reliabilities (CR) = 0.94 and average variance extracted (AVE) = 0.63, whereas the PBGEC-N had a Cronbach’s *α* = 0.93 (*p* < 0.001), CR = 0.94 and AVE = 0.61. In Sample 2, the full-scale Cronbach’s *α* was 0.83 (*p* < 0.001); the PBGEC-P scale had a Cronbach’s *α* = 0.94 (*p* < 0.001), CR = 0.95 and AVE = 0.65, whereas the PBGEC-N scale had a Cronbach’s *α* = 0.89 (*p* < 0.001), CR = 0.91 and AVE = 0.50.

The test–retest reliability for the total scale of Item-39 and subscales was as follows: total PBGEC *r* = 0.584 (*p* < 0.001), positive PBGEC *r* = 0.665 (*p* < 0.001), and negative PBGEC *r* = 0.649 (*p* < 0.001); the total scale of Item-20 and subscales was as follows: total PBGEC *r* = 0.600 (*p* < 0.001), positive PBGEC *r* = 0.676 (*p* < 0.001), and negative PBGEC *r* = 0.660 (*p* < 0.001).

#### External Validity

Gender, age, and SES (i.e., education level and household income) were used as indicators of the external validity of the PBGECS-20. In order to support Hypothesis 2, we compared PBGEC-P and PBGEC-N scores according to gender, age, education level, and household income. The following results were obtained. There were no significant differences between men and women in either the PBGEC-P (*t* = 0.09, *p* = 0.93) or PBGEC-N scores (*t* = 0.61, *p* = 0.54). There was a significant difference in PBGEC-P scores between different age groups, *F* (4,1378) = 5.71, *p* < 0.001, *η*^2^ = 0.02. *Post-hoc* tests (LSD) revealed that individuals under the age of 25 scored significantly higher than those over the age of 36–55, *p*s < 0.001. Furthermore, those aged 26–35 years also scored significantly higher than the age of 46–55, *p*s < 0.05. No significant age differences emerged for PBGEC-N scores, *F* (4,1378) = 0.86, *p* = 0.49, *η*^2^ = 0.00, as shown in Table 6.

**Table 6:** Sociodemographic characteristics on positive and negative PBGEC scores in different groups.

Topic	Sample size (*n*)	PBGEC-P (M ± SD)	*t/F*	*p*	PBGEC-N (M ± SD)	*t/F*	*p*
**Gender**							
Male	656	20.77 ± 7.68	0.09	0.93	15.33 ± 7.93	0.61	0.54
Female	638	20.73 ± 6.98			15.07 ± 7.45		
Not provided	114						
**Aged**							
17–25	386	21.95 ± 6.65	5.71^***^	0.000	15.51 ± 7.49	0.86	0.49
26–35	421	21.30 ± 7.61			15.47 ± 7.70		
36–45	353	19.94 ± 7.75			14.71 ± 7.87		
46–55	191	19.46 ± 7.55			14.90 ± 7.44		
More than 56	32	19.97 ± 6.09			16.19 ± 7.06		
Not provided	25						
**Educational level**							
JH	139	20.33 ± 8.57	1.23	0.30	13.86 ± 8.45	5.69^***^	0.000
H/T	257	21.04 ± 7.51			14.43 ± 7.93		
C	321	20.37 ± 7.92			14.42 ± 7.54		
U	581	20.99 ± 6.66			16.23 ± 7.30		
M & D	106	22.00 ± 7.99			16.21 ± 7.74		
Not provided	4						
**Household income**							
Less than 10	116	19.82 ± 8.26	5.25^***^	0.000	15.47 ± 8.82	1.71	0.10
10–20	151	20.43 ± 7.67			14.85 ± 7.84		
20–40	323	21.37 ± 7.22			15.57 ± 7.42		
40–60	325	19.54 ± 7.39			15.91 ± 7.31		
60–100	246	21.22 ± 7.17			15.00 ± 7.54		
100–150	111	22.19 ± 6.99			14.29 ± 7.42		
150–200	70	23.84 ± 6.65			12.97 ± 7.22		
More than 200	59	22.53 ± 7.24			14.75 ± 9.11		
Not provided	7						

*N_sample 1_ = 1,408. PBGEC, perceived broad group emotional climate; Educational level: JH, Junior high school level or below; H/T, high school/technical secondary school level; C, college level; U, university level; M & D, Master’s degree and doctor’s degree level. Household income:1,000 RMB is the unit of measurement*.

*** *p < 0.001*.

There were no significant differences in PBGEC-P scores based on education level [*F* (4,1399) = 1.23, *p* = 0.30, *η*^2^ = 0.00], but there were significant differences in PBGEC-N scores, *F* (4,1399) = 5.69, *p* < 0.000, *η*^2^ = 0.02. *Post-hoc* tests (LSD) revealed that those with an undergraduate and graduate education scored significantly higher than all other groups, *p*s < 0.05, as shown in Table 6.

The PBGEC-P scores differed as a function of household income, *F* (7,1393) = 5.25, *p* < 0.000, *η*^2^ = 0.03. *Post-hoc* tests (LSD) revealed that individuals with household incomes of 20,000–40,000 RMB, 60,000–100,000 RMB, 100,000–150,000 RMB, or over 200,000 RMB scored significantly higher than individuals with household incomes under 10,000 RMB or 40,000–60,000 RMB, *p*s < 0.05. Individuals with a household income of 150,000–200,000 RMB scored higher than individuals with household incomes of 10,000–20,000 RMB, 20,000–40,000 RMB, 40,000–60,000 RMB, or 60,000–100,000 RMB, *p*s < 0.05. PBGEC-N scores did not differ based on household income, *F* (7,1393) = 1.71, *p* = 0.10, *η*^2^ = 0.00 (see Table 6).

#### Criterion Validity

We predicted that the positive and negative dimensions of the PBGEC would be related to present social attitude, future social expectations, and social wellbeing (Hypotheses 3 and 4). As shown in Table 7, the PBGEC-P was positively associated with all social wellbeing dimensions (*r*s = 0.24 to 0.37), while the PBGEC-N was negatively associated with all social wellbeing dimensions (*r*s = −0.39 to −0.27). The PBGEC-P was also positively associated with positive present social attitude and positive future social expectations (*r* = 0.33 and 0.22, respectively). The PBGEC-N was positively associated with negative present social attitude and negative future social expectations (*r* = 0.26 and 0.21, respectively), as well as negatively associated with positive present social attitude and positive future social expectations (*r* = −0.38 and −0.24, respectively).

**Table 7:** Correlations of the PBGECS with present social attitude, future social expectations, social well-being.

S. No.		M	SD	1	2	3	4	5	6	7	8	9	10	11
1.	PBGEC-P	20.88	7.43	1										
2.	PBGEC-N	15.23	7.68	−0.24^***^	1									
3.	Social acceptance	16.22	3.00	0.35^***^	−0.31^***^	1								
4.	Social integration	16.65	3.53	0.37^***^	−0.39^***^	0.69^***^	1							
5.	Social contribution	22.13	3.78	0.24^***^	−0.27^***^	0.64^***^	0.67^***^	1						
6.	Social actualization	17.66	3.30	0.31^***^	−0.35^***^	0.64^***^	0.76^***^	0.79^***^	1					
7.	Social coherence	21.39	3.95	0.29^***^	−0.28^***^	0.69^***^	0.66^***^	0.79^***^	0.73^***^	1				
8.	Social well-being	94.05	15.39	0.35^***^	−0.36^***^	0.83^***^	0.86^***^	0.90^***^	0.89^***^	0.89^***^	1			
9.	Positive present social attitude	20.62	3.78	0.33^***^	−0.37^***^	0.61^***^	0.66^***^	0.57^***^	0.67^***^	0.58^***^	0.70^***^	1		
10.	Negative present social attitude	19.64	3.90	−0.03	0.26^***^	0.06^*^	−0.08^**^	0.13^**^	0.01	0.11^***^	0.05^*^	−0.00	1	
11.	Positive future social expectation	8.89	1.79	0.22^***^	−0.24^***^	0.46^***^	0.56^***^	0.61^***^	0.69^***^	0.55^***^	0.66^***^	0.63^***^	0.07^*^	1
12.	Negative future social expectation	7.23	2.04	0.02	0.21^***^	0.07^*^	−0.11^***^	−0.03	−0.11^***^	0.02	−0.04	−0.03	0.56^***^	−0.10^***^

*N_sample 1_ = 1,408. PBGEC, perceived broad group emotional climate; PBGEC-P, positive perceived broad group emotional climate; PBGEC-N, negative perceived broad group emotional climate*.

*** *p < 0.001*,

** *p < 0.01*,

* *p < 0.05*.

## Discussion

This research developed a PBGECS for measuring how individuals evaluate the emotional climate of the people around them. Based on a literature review and a series of studies, this research shows that the scale of PBGEC-20 has strong reliability and validity. Our results suggest that our developed scale performs effectively.

First, we clarified that PBGEC and individual emotion can be considered separate constructs. Our delineation aligns with the existing presumption that although individual emotion and PBGEC are based on the same psychological system, the two are still conceptually independent (Rahn et al., 1996). Next, Samples 1 and 2 in the present study were also employed to determine whether PBGEC and individual emotion could together form four-factor model: PBGEC-P, PBGEC-N, PA, and NA. Notably, this four-factor model had a better fit than the two-factor model or one-factor model. Hence, Hypotheses 1 and 2 were also supported.

Second, PBGECS has strong validity. We examined the scale’s construct validity, cultural validity, population validity, and interpretive validity (Washington and McLoyd, 1982). For construct validity, the PBGEC-20 can be divided into positive and negative dimensions along with its correlated two-factor model. It is consistent with the PANAS’ dimensions and structures (Seib-Pfeifer et al., 2017). For cultural validity, PBGEC-P was positively associated with positive present social attitude and positive future social expectations, while PBGEC-N was negatively associated with positive present social attitude and positive future social expectations, as well as positively associated with negative present social attitude and negative future social expectations. For population validity, PBGEC-P was positively associated with all five social wellbeing dimensions, while PBGEC-N was negatively correlated with social wellbeing. For interpretive validity, PBGEC-P was highest among individuals under the age of 35 and those with higher household incomes, while PBGEC-N scores were highest among more educated participants. Our findings are consistent with the fact that SES best predicts familial emotional climate (Farrell et al., 2018). These results align with Hypothesis 3, Hypothesis 3A, Hypothesis 4, and Hypothesis 5.

The present study also revealed that there is an important dynamic quality to PBGEC that generates not group-based emotions, but general group emotions. Specifically, this study is similar to Kuppens and Yzerbyt’s (2014) research indicating that PBGEC-P has a positive correlation with social identification; and PBGEC-N has a negative correlation with social identification. At the same time, the results of this research provided an empirical evidence for the study of Kuppens and Yzerbyt.

The scale can be used for research to determine how individuals perceive and experience of the emotion climate when interacting with group members in daily life. Future research may use this scale to determine the relationship between individuals’ perceived emotional climate in their surroundings and other variables, including social adaptation behavior, interpersonal relationships, and relative deprivation.

### Implications and Connections to Past Research

Perceived broad group emotional climate represents group emotions as perceived by individuals (Marraccini et al., 2020), which may in turn influence an individual’s attitudes and societal functions (De Rivera and Páez, 2007). For example, a positive perception of broad group emotional climate, as opposed to a negative perception, has an orientation toward the future and is associated with greater wellbeing. According to the social verification and self-categorization theory, such affects are powerful psychological foundations upon which individuals come to understand reality (Shteynberg, 2009). While the PBGEC is important for an individual’s daily life, it is relatively difficult to turn it into a precise concept that can be objectively measured (De Rivera, 1992). Through previous studies, we know that emotional climate is slightly different from PBGEC and that only emotional climate scales currently exist. Given this, it is necessary to develop a PBGECS to measure individuals’ perceptions and experiences of broad group emotions.

This study advances our knowledge of emotional climate by offering a measure of PBGEC. Our scale was developed in response to Rousseau’s (1988) appeal for the development of a scale that measures specific emotional climates (Liu et al., 2014). Our scale was based on general group emotions in China and enriches existing research sampled from Western cultural groups. We differentiated concepts related to group emotional climate and viewed individuals’ subjective experience as the most important aspect that should be considered.

Each era has its own method for determining appropriate display rules for emotions and expressions. The present study investigated individual PBGEC by constructing an emotion lexicon suited for group emotions that was based on psychological emotion models. PBGEC plays an important role in influencing people’s moral and social life (Zhou and Yu, 2015) and is ubiquitous in people’s daily lives, not only for individual behavior, but also for intra-group relationships and social stability (Wang and Wang, 2009; Kuppens, 2011).

Previous emotional climate studies have divided this construct into positive and negative climate. Prior work predominantly focused on the domains of commerce, politics, economics, and communication. By studying PBGEC within individuals’ daily lives, the present study suggests that individuals may engage in positive evaluations of the world when in a good emotional climate, and engage in negative evaluations when in a bad emotional climate (Rahn et al., 1996). Therefore, our study supports the conclusion that individuals’ present social attitudes and future social expectations may be positive when in a PBGEC-P and vice versa. The resulting perceptions may affect people’s attitudes, motivations, and behaviors (De Rivera, 1992). Specifically, a positive emotional climate influences a range of psychosocial phenomena, such as decision-making, tolerance toward diversity, willingness to build cooperation, and social unity. Conversely, a negative emotional climate tends to hobble interpersonal harmony and contribute to interpersonal conflicts and repressive decisions (see De Rivera, 1992; Conejero and Etxebarria, 2007; De Rivera et al., 2007; De Rivera and Páez, 2007; Pelletier, 2018).

In addition, individual social emotion (i.e., PBGEC of this study) is an important part of people’s social mentality and an important reflection of a country’s social governance (Wang et al., 2007; Wang, 2013a,b, 2018). The PBGECS assesses the perceived emotion climate of an individual’s surrounding environment, which may also can help government personnel understand people’s social mentality and serve as a public opinion barometer of the government’s governance effect, so as to promote good governance and improve people’s wellbeing.

### Limitations and Future Directions

A few study limitations should be noted. First, the current PBGECS only applies to the Chinese context for Chinese community residents and university students. Due to constraints regarding research staff, available materials, and financial resources, we could only examine participants from the southwestern and central regions of China. Future research should include samples from other regions and other cultures to increase the representativeness of the sample and enhance the generalizability of the present findings. Second, we chose not to use the “arousal” dimension in the structure of PBGEC after taking the context of China, which is a collectivist culture into consideration. Some evidence in the scientific literature notes that the emotional display rules and expressions differ between those from Asia and those from Western cultures (Liu et al., 2014). For example, emotions of positive and negative valence tend to be associated with increased arousal in Western cultures, whereas valence and arousal tend to be experienced relatively independently from each other in East Asian cultures (Kuppens et al., 2013). In other words, high arousal positive affect is preferred in Western cultures and lower arousal positive affect is preferred in East Asian cultures (Tsai et al., 2006). Third, our study only explored PBGEC at the individual level. In the future, it would be necessary to explore PBGEC at the group level using the other types of group emotion. Finally, there are many other factors that may affect PBGEC, such as violence and apologies, among others (Bobowik et al., 2017). The present study only examined SES as the predicting variable. Future research should use other, more influence factors to study PBGEC. Certainly, according to the nomological validity, we can also consider those variables which are low relate or no relate to PBGEC, such as personality factors, in order to investigate the nomological validity of PBGEC.

Future research can consider the potential clinical application of PBGEC in the important work of general group emotion in clinical intervention. For example, during the COVID-19 pandemic, the influence of people around on the health of patients and whether remote psychotherapy can be adopted to effectively improve the individual’s perception of the emotional environment (Poletti et al., 2020).

## Conclusion

Perceived broad group emotional climate and individual emotion are two related but distinct concepts. The PBGECS scale devised from the present analyses revealed a two-dimensional structure, consisting of PBGEC-P and PBGEC-N. The correlated two-factor model demonstrated the best fit to the data. Together, the two (i.e., positive and negative) form four dimensions: PBGEC-P, PBGEC-N, PA, and NA. Our results indicate that SES affects PBGEC. The PBGEC was significantly correlated with present social attitudes, future social expectations, and social wellbeing. Finally, the PBGECS shows satisfactory reliability and validity in the Chinese context for Chinese community residents and university students. Thus, the PBGECS can be used as an effective and reliable tool for quantifying PBGEC. In the future research, this scale can provide as psychosocial context information that affect behavior. In turn, researchers can benefit from the PBGECS to predict and explain some behaviors (e.g., collective behavior and individual social adaptive behavior).

## Data Availability Statement

The datasets presented in this study can be found in online repositories. The names of the repository/repositories and accession number(s) can be found at: https://osf.io/gh3az/.

## Ethics Statement

The studies involving human participants were reviewed and approved by the Ethics Committee of School of Psychology, Jiangxi Normal University. Written informed consent to participate in this study was provided by the participants’ legal guardian/next of kin.

## Author Contributions

XY and XW: research framework design, data collation and analysis, and writing and revising the paper. XW: The development of PBGECS. JL, SL, ML, and BY: revising the paper. XY and XW contributed equally to this work and should be considered co-first authors. All authors contributed to the article and approved the submitted version.

## Conflict of Interest

The authors declare that the research was conducted in the absence of any commercial or financial relationships that could be construed as a potential conflict of interest.

## Publisher’s Note

All claims expressed in this article are solely those of the authors and do not necessarily represent those of their affiliated organizations, or those of the publisher, the editors and the reviewers. Any product that may be evaluated in this article, or claim that may be made by its manufacturer, is not guaranteed or endorsed by the publisher.

## References

[ref1] Altheide D. L. Johnson J. M. (1994). “Criteria for assessing interpretive validity in qualitative research,” in Handbook of Qualitative Research. eds. Denzin N. K. Lincoln Y. S. Newbury Park, CA: Sage, 485–499.

[ref2] Atkinson J. W. Cartwright D. (1964). Some neglected variables in contemporary conceptions of decision and performance. Psychol. Rep. 14, 575–590. 10.2466/pr0.1964.14.2.575

[ref3] Bakhtiar A. Webster E. A. Hadwin A. F. (2018). Regulation and socio-emotional interactions in a positive and a negative group climate. Metacogn. Learn. 13, 57–90. 10.1007/s11409-017-9178-x

[ref4] Barrett L. F. Russell J. A. (1999). The structure of current affect: controversies and emerging consensus. Curr. Dir. Psychol. Sci. 8, 10–14. 10.1111/1467-8721.00003

[ref5] Barsade S. G. (2002). The ripple effect: emotional contagion and its influence on group behavior. Adm. Sci. Q. 47, 644–675. 10.2307/3094912

[ref6] Barsade S. G. Gibson D. E. (1998). “Group emotion: a view from top and bottom,” in Research on Managing Groups and Teams. *Vol*. 1, ed. Gruenfeld and Colleagues D. H. Stamford, CT: JAI Press, 81–102.

[ref7] Barsade S. G. Knight A. P. (2015). Group affect. Annu. Rev. Organ. Psych. Organ. Behav. 2, 21–46. 10.1146/annurev-orgpsych-032414-111316

[ref8] Bar-Tal D. (2007). Sociopsychological foundations of intractable conflicts. Am. Behav. Sci. 50, 1430–1453. 10.1177/0002764207302462

[ref9] Beedie C. Terry P. Lane A. (2005). Distinctions between emotion and mood. Cognit. Emot. 19, 847–878. 10.1080/02699930541000057

[ref10] Bobowik M. Páez D. Arnoso M. Cárdenas M. Rimé B. Zubieta E.. (2017). Institutional apologies and socio-emotional climate in the south American context. Br. J. Soc. Psychol. 56, 578–598. 10.1111/bjso.12200 28547845

[ref11] Caporael L. R. Baron R. M. (1997). “Groups as the mind’s natural environment,” in Evolutionary Social Psychology. eds. Simpson J. Kenrick D. (Mahwah, NJ: Lawrence Erlbaum Associates, Inc), 317–343.

[ref12] Chen X. Y. Wang X. Q. Liu J. P. Dong S. H. Zhu J. C. Huo J. Y. (2018). Effects of relative deprivation on intention to rebel: a multiple mediation model. J. Pac. Rim Psychol. 12, 1–10. 10.1017/prp.2017.25

[ref13] Collins A. L. Lawrence S. A. Troth A. C. Jordan P. J. (2013). Group affective tone: a review and future research directions. J. Organ. Behav. 34(Suppl. 1), S43–S62. 10.1002/job.1887

[ref14] Conejero S. Etxebarria I. (2007). The impact of the Madrid bombing on personal emotions, emotional atmosphere and emotional climate. J. Soc. Issues 63, 273–287. 10.1111/j.1540-4560.2007.00508.x

[ref15] Cook J. Bird G. (2011). Social attitudes differentially modulate imitation in adolescents and adults. Exp. Brain Res. 211, 601–612. 10.1007/s00221-011-2584-4, PMID: 21336831 PMC3102210

[ref16] Cronbach L. J. Meehl P. E. (1955). Construct validity in psychological tests. Psychol. Bull. 52, 281–302. 10.1037/h0040957, PMID: 13245896

[ref17] De Rivera J. (1977). A structural theory of the emotions. Psychol. Issues 10, 98–129. PMID: 887708

[ref18] De Rivera J. (1992). “Emotional climate: Social structure and emotional dynamics,” in International Review of Studies on Emotion, *Vol*. 2. ed. Strongman K. T., John Wiley & Sons Ltd, 197–218.

[ref19] De Rivera J. Kurrien R. Olsen N. (2007). The emotional climate of nations and their culture of peace. J. Soc. Issues 63, 255–271. 10.1111/j.1540-4560.2007.00507.x

[ref20] De Rivera J. Páez D. (2007). Emotional climate, human security, and cultures of peace. J. Soc. Issues 63, 233–253. 10.1111/j.1540-4560.2007.00506.x

[ref21] Esses V. M. Dovidio J. F. (2002). The role of affect in determining willingness to engage in intergroup contact. Personal. Soc. Psychol. Bull. 28, 1202–1214. 10.1177/01461672022812006

[ref22] Farrell A. K. Slatcher R. B. Tobin E. T. Imami L. Wildman D. E. Luca F.. (2018). Socioeconomic status, family negative emotional climate, and anti-inflammatory gene expression among youth with asthma. Psychoneuroendocrinology 91, 62–67. 10.1016/j.psyneuen.2018.02.011, PMID: 29529520 PMC5903571

[ref23] Florin P. Giamartino G. A. Kenny D. A. Wandersman A. (1990). Levels of analysis and effects: clarifying group influence and climate by separating individual and group effects. J. Appl. Soc. Psychol. 20, 881–900. 10.1111/j.1559-1816.1990.tb01466.x

[ref24] Forgas J. P. (1995). Mood and judgment: the affect infusion model (AIM). Psychol. Bull. 117, 39–66. 10.1037/0033-2909.117.1.39, PMID: 7870863

[ref25] Forgas J. P. (2002). Feeling and doing: affective influences on interpersonal behavior. Psychol. Inq. 13, 1–28. 10.1207/s15327965pli1301_01

[ref26] Frijda N. H. Mesquita B. (1994). “The social roles and functions of emotions,” in Emotion and Culture. eds. Kitayama S. Markus H. (New York: American Psychological Association), 51–87.

[ref27] Gamero N. González-Romá V. Peiró J. M. (2008). The influence of intra-team conflict on work teams’ affective climate: a longitudinal study. J. Occup. Organ. Psychol. 81, 47–69. 10.1348/096317907X180441

[ref28] Gaudreau P. Sanchez X. Blondin J. P. (2006). Positive and negative affective states in a performance-related setting: testing the factorial structure of the PANAS across two samples of French-Canadian participants. Eur. J. Psychol. Assess. 22, 240–249. 10.1027/1015-5759.22.4.240

[ref29] George J. M. (1990). Personality, affect, and behavior in groups. J. Appl. Psychol. 75, 107–116. 10.1037/0021-9010.75.2.107

[ref30] George J. M. (1996). “Group affective tone,” in Handbook of Workgroup Psychology. ed. West M. A. (Chichester, UK: John Wiley & Sons Ltd), 77–93.

[ref31] Hu Z. F. Mo L. (2002). On the integration of factor analysis methods. J. Psychol. Sci. 25, 474–475. 10.16719/j.cnki.1671-6981.2002.04.028

[ref32] Huang L. Yang T. Z. Ji Z. M. (2003). Applicability of the positive and negative affect scale in Chinese. Chin. Ment. Health J. 1, 54–56. 10.3321/j.issn:1000-6729.2003.01.018

[ref33] Ilies R. Wagner D. T. Morgeson F. P. (2007). Explaining affective linkages in teams: individual differences in susceptibility to contagion and individualism-collectivism. J. Appl. Psychol. 92, 1140–1148. 10.1037/0021-9010.92.4.1140 17638471

[ref34] Kelly J. R. Barsade S. G. (2001). Mood and emotions in small groups and work teams. Organ. Behav. Hum. Decis. Process. 86, 99–130. 10.1006/obhd.2001.2974

[ref35] Keyes C. L. M. (1998). Social well-being. Soc. Psychol. Q. 61, 121–140. 10.2307/2787065

[ref36] Knight A. P. Eisenkraft N. (2015). Positive is usually good, negative is not always bad: the effects of group affect on social integration and task performance. J. Appl. Psychol. 100, 1214–1227. 10.1037/apl0000006 25495091

[ref37] Kuppens T. (2011). Feeling like a group member: Social identity, group-based appraisals and group-based emotions. dissertation. Université Catholique de Louvain.

[ref38] Kuppens P. Tuerlinckx F. Russell J. A. Barrett L. F. (2013). The relation between valence and arousal in subjective experience. Psychol. Bull. 139, 917–940. 10.1037/a0030811, PMID: 23231533

[ref39] Kuppens T. Yzerbyt V. Y. (2014). When are emotions related to group-based appraisals? A comparison between group-based emotions and general group emotions. Personality Social Psychol. Bull. 40, 1574–1588. 10.1177/0146167214551542 25260364

[ref40] Lai A. T. (2013). The transmission route of group emotion. doctoral dissertation. Capital Normal University.

[ref41] Liu X. Y. Härtel C. E. J. Sun J. J. M. (2014). The workgroup emotional climate scale: theoretical development, empirical validation, and relationship with workgroup effectiveness. Group Org. Manag. 39, 626–663. 10.1177/1059601114554453

[ref42] Liu X. L. Wang J. X. (2020). The influence of the socioeconomic status and the subjective social status on one’s well-being: an empirical analysis based on CGSS (2010–2015). J. Guangxi Normal Univ. 56, 14–27. 10.16088/j.issn.1001-6597.2020.05.002

[ref43] Lu M. Hamamura T. Doosje B. Suzuki S. Takemura K. (2016). Culture and group-based emotions: could group-based emotions be dialectical? Cognit. Emot. 31, 937–949. 10.1080/02699931.2016.1185394, PMID: 27224204

[ref44] Ma A. (2010). The Characteristic of Social Attitude of Chinese People. Beijing: China University of Politic Science and Law Press, 15–26. 81–93.

[ref45] Mackie D. M. Smith E. R. (2017). Group-based emotion in group processes and intergroup relations. Group Process. Intergroup Relat. 20, 658–668. 10.1177/1368430217702725

[ref46] Mahdavian M. Safizadeh H. (2015). Measurement of socioeconomic status in Iran: a systematic review. Asian J. Agric. Extension Econ. Sociol. 6, 1–15. 10.9734/AJAEES/2015/16756

[ref47] Marraccini M. E. Fang Y. Levine S. P. Chin A. J. Pittleman C. (2020). Measuring student perceptions of school climate: a systematic review and ecological content analysis. Sch. Ment. Heal. 12, 195–221. 10.1007/s12310-019-09348-8

[ref48] Miller D. A. Smith E. R. Mackie D. M. (2004). Effects of intergroup contact and political predispositions on prejudice: role of intergroup emotions. Group Process. Intergroup Relat. 7, 221–237. 10.1177/1368430204046109

[ref49] Moons W. G. Leonard D. J. Mackie D. M. Smith E. R. (2009). I feel our pain: antecedents and consequences of emotional self-stereotyping. J. Exp. Soc. Psychol. 45, 760–769. 10.1016/j.jesp.2009.04.016

[ref50] Mueller C. W. Parcel T. L. (1981). Measures of socioeconomic status: alternatives and recommendations. Child Dev. 52, 13–30. 10.2307/1129211

[ref51] Murdock K. W. LeRoy A. S. Fagundes C. P. (2017). Early-life socio-economic status and adult health: the role of positive affect. Stress. Health 33, 190–198. 10.1002/smi.2696, PMID: 27443423 PMC5253328

[ref52] Muthén L. K. Muthén B. O. (1998–2012). Mplus User’s Guide. 7th Edn. Los Angeles, CA: Muthén & Muthén.

[ref53] Niedenthal P. M. Brauer M. (2012). Social functionality of human emotion. Annu. Rev. Psychol. 63, 259–285. 10.1146/annurev.psych.121208.131605, PMID: 22017377

[ref54] Nurmi J. E. (1991). How do adolescents see their future? A review of the development of future orientation and planning. Dev. Rev. 11, 1–59. 10.1016/0273-2297(91)90002-6

[ref55] Páez D. Espinosa A. Bobowik M. (2013). “Emotional climate: how is it shaped, fostered, and changed?” in Changing Emotions. eds. Hermans D. B. Rimé Mesquita B. (London: Psychology Press), 113–119.

[ref56] Paquette D. Ryan J. (2001). Bronfenbrenner’s ecological systems theory. Retrieved from http://people.usd.edu/~mremund/bronfa.pdf

[ref57] Pelletier P. (2018). The pivotal role of perceived emotional synchrony in the context of terrorism: challenges and lessons learned from the March 2016 attack in Belgium. J. Appl. Soc. Psychol. 48, 477–487. 10.1111/jasp.12526

[ref58] Poletti B. Tagini S. Brugnera A. Parolin L. Pievani L. Ferrucci R.. (2020). Telepsychotherapy: a leaflet for psychotherapists in the age of COVID-19. A review of the evidence. Couns. Psychol. Q., 1–16. 10.1080/09515070.2020.1769557, [Epub ahead of print]

[ref59] Rahn W. M. Kroeger B. Kite C. M. (1996). A framework for the study of public mood. Polit. Psychol. 17, 29–58. 10.2307/3791942

[ref60] Rousseau D. M. (1988). “The construction of climate in organization research,” in International Review of Industrial and Organizational Psychology. eds. Cooper C. L. Robertson I. T. (Chichester, UK: John Wiley & Sons Ltd), 139–159.

[ref61] Ruiz J. I. (2007). Emotional climate in organizations: applications in Latin American prisons. J. Soc. Issues 63, 289–306. 10.1111/j.1540-4560.2007.00509.x

[ref62] Russell J. A. (1980). A circumplex model of affect. J. Pers. Soc. Psychol. 39, 1161–1178. 10.1037/h0077714

[ref63] Salami T. K. Walker R. L. (2014). Socioeconomic status and symptoms of depression and anxiety in African American college students: the mediating role of hopelessness. J. Black Psychol. 40, 275–290. 10.1177/0095798413486158

[ref64] Schneider B. Reichers A. E. (1983). On the etiology of climates. Pers. Psychol. 36, 19–39. 10.1111/j.1744-6570.1983.tb00500.x

[ref65] Seib-Pfeifer L. Pugnaghi G. Beauducel A. Leue A. (2017). On the replication of factor structures of the positive and negative affect schedule (PANAS). Personal. Individ. Differ. 107, 201–207. 10.1016/j.paid.2016.11.053

[ref66] Shteynberg G. (2009). Social attention theory: A new look at knowledge formation in groups. Doctoral dissertation. University of Maryland.

[ref67] Smith E. R. Seger C. R. Mackie D. M. (2007). Can emotions be truly group level? Evidence regarding four conceptual criteria. J. Pers. Soc. Psychol. 93, 431–446. 10.1037/0022-3514.93.3.431, PMID: 17723058

[ref68] Totterdell P. (2000). Catching moods and hitting runs: mood linkage and subjective performance in professional sport teams. J. Appl. Psychol. 85, 848–859. 10.1037/0021-9010.85.6.848 11125650

[ref69] Totterdell P. Kellett S. Briner R. B. Teuchmann K. (1998). Evidence of mood linkage in work groups. J. Pers. Soc. Psychol. 74, 1504–1515. 10.1037/0022-3514.74.6.1504

[ref70] Tsai J. L. Knutson B. Fung H. H. (2006). Cultural variation in affect valuation. J. Pers. Soc. Psychol. 90, 288–307. 10.1037/0022-3514.90.2.288, PMID: 16536652

[ref71] Wang J. X. (2011). Paying attention to people’s dignity and well-being to promote social justice and harmony – a study on social mentality in China from 2010 to 2011. Democracy Sci. (3), 68–73.

[ref72] Wang J. X. (2013a). Social emotions in the perspective of social mentality. J. Yunnan Normal Univ. 45, 55–63.

[ref73] Wang J. X. (2013b). Paying attention to social emotions, promoting social identity and cohesion of social consensus – a study on social mentality in China from 2012 to 2013. Democracy Sci. 64–71. 10.3969/j.issn.1003-0026.2013.01.022

[ref74] Wang J. X. (2013c). Actively gather the positive energy of social mentality. China’s National Conditions and National Strength, (5), 13–15. 10.13561/j.cnki.zggqgl.2013.05.015

[ref75] Wang J. X. (2018). The social mentality of different subjective social strata. Jiangsu Social Sci. 24–33. 10.13858/j.cnki.cn32-1312/c.2018.01.005

[ref76] Wang Z. J. Hou Y. R. Tang H. Y. Zhao Z. Z. Chen H. X. (2017). The amplification effect of group-shared emotion. Adv. Psychol. Sci. 25, 662–671. 10.3724/SP.J.1042.2017.00662

[ref77] Wang J. Y. Wang C. J. (2009). The investigation and analysis of minority social sentiment in the northwest poverty-stricken areas. Gansu Social Sci. 4, 30–33. 10.15891/j.cnki.cn62-1093/c.2009.04.049

[ref78] Wang J. X. Yang Y. Y. Chen W. Q. (2007). Chinese social mentality survey report. Democracy Sci. 2, 40–44.

[ref79] Washington E. D. McLoyd V. C. (1982). The external validity of research involving American minorities. Hum. Dev. 25, 324–339. 10.1159/000272817

[ref80] Watson D. Clark L. A. Tellegen A. (1988). Development and validation of brief measures of positive and negative affect: the PANAS scales. J. Pers. Soc. Psychol. 54, 1063–1070. 10.1037/0022-3514.54.6.1063 3397865

[ref81] Watson D. Tellegen A. (1985). Toward a consensual structure of mood. Psychol. Bull. 98, 219–235. 10.1037/0033-2909.98.2.219, PMID: 3901060

[ref82] Watson D. Wiese D. Vaidya J. Tellegen A. (1999). The two general activation systems of affect: structural findings, evolutionary considerations, and psychobiological evidence. J. Pers. Soc. Psychol. 76, 820–838. 10.1037/0022-3514.76.5.820

[ref83] Yik M. S. M. Russell J. A. (2003). Chinese affect circumplex: I. Structure of recalled momentary affect. Asian J. Soc. Psychol. 6, 185–200. 10.1046/j.1467-839X.2003.00120.x

[ref84] Zhao X. (2010). A study on the compile of the questionnaire for the social well-being of college students and its relationship with their parental rearing pattern. master’s thesis. Southwest University.

[ref85] Zhou X. L. Yu H. B. (2015). The brain basis of social emotion and social behavior. J. Suzhou Univ. 1, 37–47. 10.19563/j.cnki.sdjk.2015.01.006

