# Supplementary material for: The Perceived Broad Group Emotional Climate Scale: Development and Validation With Chinese Community Residents and University Students
Source: Front Psychol. 2021 Aug 25;12:686734. doi: 10.3389/fpsyg.2021.686734 (PMC8424200; doi:10.3389/fpsyg.2021.686734)
Supplement: Supplementary file 1 [file Data_Sheet_1.doc]

**Appendix A**

**1.The Perceived Broad Group Emotional Climates Scale-20 items (PBGECS-20)**

**1.1The subscale of positive PBGEC**

**您认为当前你身边居民在多大程度上体验到了这10个形容词所描述的积极情绪情感**(哪一数值最接近左边或右边词的情况)**：**

To what extent do you think the people around you (such as your community residents ) experience the emotions described by the following 10 positive words? (which value is closest to the words on the left or right) :

| 题目  Item | 完全没有  Not at all | 有些  Some | 中等程度  Medium | 较多  More | 非常强烈  Very strongly |
| --- | --- | --- | --- | --- | --- |
| 1. 愉悦（Cheerful) | 0 | 1 | 2 | 3 | 4 |
| 2. 热情(Enthusiastic) | 0 | 1 | 2 | 3 | 4 |
| 3. 开心(Happy) | 0 | 1 | 2 | 3 | 4 |
| 4. 享受(Enjoyable) | 0 | 1 | 2 | 3 | 4 |
| 5. 兴奋(Excited) | 0 | 1 | 2 | 3 | 4 |
| 6. 活跃(Vibrant) | 0 | 1 | 2 | 3 | 4 |
| 7. 鼓舞(Inspiring) | 0 | 1 | 2 | 3 | 4 |
| 8. 自豪(Proud) | 0 | 1 | 2 | 3 | 4 |
| 9. 欣喜(Delighted) | 0 | 1 | 2 | 3 | 4 |
| 10. 满意(Satisfied) | 0 | 1 | 2 | 3 | 4 |

**1.2 The subscale of negative PBGEC**

**您认为当前你身边居民在多大程度上体验到了这10个形容词所描述的消极情绪情感**(哪一数值最接近左边或右边词的情况)：

To what extent do you think the people around you (such as your community residents ) experience the emotions described by the following 10 negative words? (which value is closest to the words on the left or right) :

| **题目**  Item | **完全没有**  Not at all | **有些**  Some | **中等程度**  Medium | **较多**  More | **非常强烈**  Very strongly |
| --- | --- | --- | --- | --- | --- |
| 1. 焦虑(Anxious) | 0 | 1 | 2 | 3 | 4 |
| 2. 担忧(Worried) | 0 | 1 | 2 | 3 | 4 |
| 3. 害怕(Afraid) | 0 | 1 | 2 | 3 | 4 |
| 4. 恐惧(Fearful) | 0 | 1 | 2 | 3 | 4 |
| 5. 郁闷(Distressed) | 0 | 1 | 2 | 3 | 4 |
| 6. 冷漠(Indifferent) | 0 | 1 | 2 | 3 | 4 |
| 7. 不满(Dissatisfied) | 0 | 1 | 2 | 3 | 4 |
| 8. 怨恨(Resentment) | 0 | 1 | 2 | 3 | 4 |
| 9. 痛苦(Misery) | 0 | 1 | 2 | 3 | 4 |
| 10. 贪欲(Greedy) | 0 | 1 | 2 | 3 | 4 |

1. **Notes (about the PBGECS-20 used to test college students)**

在PBGECS-20用于大学生测试时，将指导语中的“居民”修改为“居民或大学生”。

Note: When PBGECS-20 is used to test college students, the "your community residents" in the guide is changed to "your community residents or your classmates".
